# Aggressive Metastatic Insulinoma in a Patient of Diabetes Mellitus with Documentation on Dual-Tracer PET-CT ([68Ga]Ga-DOTATATE and [18F]FDG): Clinical Benefits with Combined Chemo-PRRT Approach

**DOI:** 10.1055/s-0044-1788735

**Published:** 2024-07-24

**Authors:** Yeshwanth Edamadaka, Rahul V. Parghane, Sandip Basu

**Affiliations:** 1 Radiation Medicine Centre, Bhabha Atomic Research Centre, Tata Memorial Hospital Annexe, Mumbai, Maharashtra, India; 2 Homi Bhabha National Institute, Mumbai, Maharashtra, India

**Keywords:** insulinoma, pancreatic neuroendocrine tumor, diabetes mellitus, peptide receptor radionuclide therapy, chemo-PRRT, [68Ga]Ga-DOTATATE-PET/CT and [18F]FDG-PET/CT scans

## Abstract

Insulinoma is a relatively uncommon pancreatic neuroendocrine tumor, with approximately 10% of the cases being malignant. Diabetes mellitus (DM) with concurrent insulinoma is very rare and the diagnosis of such condition is easily missed as it can be misconstrued as improved glycemic control. Therefore, persistent hypoglycemic symptoms even after stopping antidiabetic medications may be considered for insulinoma. Herein, we present a patient with DM and pancreatic insulinoma with extensive hepatic and skeletal metastases on dual-tracer positron emission tomography/computed tomography (PET/CT) ([68Ga]Ga-DOTATATE and [18F]fluorodeoxyglucose). Given the extensive disease, the patient was treated with a combination of peptide receptor radionuclide therapy (PRRT) and chemotherapy (capecitabine and temozolomide). During therapy, patient showed early clinical and imaging response for insulinoma leading to unmasking of poor glycemic control necessitating requirement of insulin administration for DM. The patient did not experience any life-threatening hypoglycemia during the chemo-PRRT treatment and showed an improvement in quality of life. Unfortunately, the disease progressed at the 4th cycle, 10 months after the initiation of PRRT. We conclude that combined chemo-PRRT may be considered an effective treatment option for patients with metastatic insulinoma and DM owing to its favorable imaging response and effective symptom control.

## Introduction

Insulinoma is a relatively rare functional pancreatic neuroendocrine tumor (panNET) with estimated incidence of four cases per million per year. Usually, it is solitary and of benign nature. About 10% of the cases are malignant, liver being the most common site of involvement. 1 The association of type 2 diabetes mellitus (DM) and insulinoma is unusual and pose a diagnostic challenge as the common cause of hypoglycemia in such patients is iatrogenic. Persistent hypoglycemic symptoms even after stopping antidiabetic medication give a subtle clue for possible insulinoma lurking within.

Treatment of malignant insulinoma using peptide receptor radionuclide therapy (PRRT) offers a promising therapeutic strategy showing an effective symptomatic control and imaging response. We herein present a rare case of concomitant metastatic insulinoma and DM, who received combined PRRT and chemotherapy (referred to as chemo-PRRT) which showed significant early clinical, biochemical, and imaging response. Unfortunately, at a later phase, the patient's condition worsened over time, and he ultimately succumbed to his disease and passed away.

## Case History

**Fig. 1:**
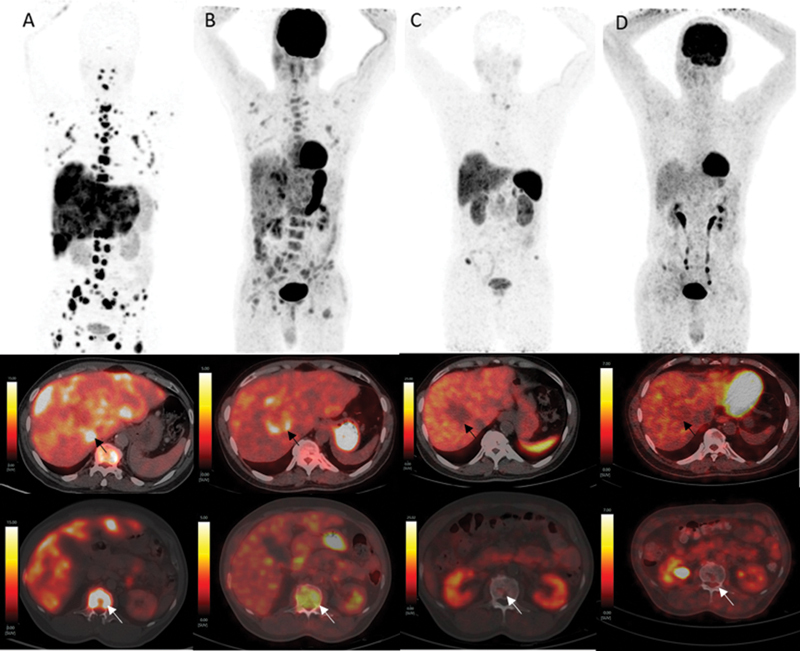
Dual-tracer positron emission tomography/computed tomography (PET/CT) ([68Ga]Ga-DOTATATE and [18F]fluorodeoxyglucose [FDG]) scans with maximum intensity projection (MIP) images showing both somatostatin receptor (SSTR) and [18F]FDG uptake in extensive liver and skeletal lesions at baseline ( **A** and **B** ). Following combined chemo-peptide receptor radionuclide therapy (PRRT) (post-2 cycles of PRRT), dual-tracer-PET/CT imaging ( **C** and **D** ) showed reduction in disease extent and SSTR uptake with significant interval resolution in metabolic activity suggestive of favorable imaging response to administered therapy. Axial fusion images (middle row) demonstrating a necrotic reference hepatic lesion (black arrow) in segment VII on [68Ga]Ga-DOTATATE scan with (maximum standardized uptake value [SUVmax] 20.6 vs. 8.7 post-2 cycles of PRRT) ( **A** and **C** , respectively) and on [18F]FDG scan with (SUVmax 6.0 vs. 3.8) ( **B** and **D** , respectively). Axial fusion images (lower row) demonstrating a lytic lesion in L1 vertebral body (white arrow) on [68Ga]Ga-DOTATATE scan with (SUVmax 26.2 vs. 3.42 post-2 cycles of PRRT) ( **A** and **C** , respectively) and on [18F]FDG scan with (SUVmax 4.46 vs. 3.2) ( **B** and **D** , respectively).

A 53-year-old male patient with known case of type 2 DM on oral antidiabetics (OAD) medications for 5 years. He presented with multiple episodes of vomiting, loss of appetite, and dyspepsia. For these symptoms, he was evaluated with contrast-enhanced computed tomography (CT) of abdomen, which showed ill-defined enhancing pancreatic body mass with multiple necrotic peripancreatic nodes and multiple bilobar liver lesions. Endoscopic ultrasound-guided pancreatic biopsy revealed well-differentiated grade I NET, MIB-1 labeling index was 2% establishing diagnosis of panNET. Patient was started on somatostatin analogs (SSAs), injection of octreotide long acting-repeatable (LAR) every month intramuscularly. Initially, patient had no hypoglycemia-related symptoms and after nine injections of SSAs, he presented with hypoglycemia (27 mg/dL), which persisted even after stopping OAD and his symptoms improved with intravenous glucose administration. He was further investigated and found to have increased insulin (33 mIU/mL) and C-peptide (6.40 ng/mL) levels with normal cortisol levels (34.95 mcg/dL) suggestive of clinical and biochemical diagnosis of insulinoma. The tumor markers of NET were significantly raised with serum chromogranin A of 44,520 ng/dL and urinary 24-hour 5-hydroxyindole acetic acid (24-h 5-HIAA) of 32.40 mg/24 hours. The patient was referred to our institute for PRRT 12 months after the initial diagnosis of panNET. At our institute, patient underwent dual-tracer positron emission tomography (PET) by using [ ^68^ Ga]Ga-DOTATATE-PET/CT and [ ^18^ F]fluorodeoxyglucose (FDG)-PET/CT scans, which showed both somatostatin receptor and [ ^18^ F]FDG expressing pancreatic primary lesion, metastatic bilobar liver lesions, and metastatic multiple axial and appendicular skeletal lesions as shown in Fig. 1 . He was then planned for a combination of chemo-PRRT therapy with [ ^177^ Lu]-Lu-DOTATATE and capecitabine and temozolomide (CAPTEM) based on dual-tracer PET/CT findings. 2 During the first two cycles of chemo-PRRT, he showed a significant biochemical response, with a reduction in serum chromogranin A to 187.40 ng/dL and 5-HIAA to 3.36 mg/24 h. The treatment response unmasked his diabetic/poor glycemic control, requiring more insulin administration. Dual-tracer PET/CT showed significant reduction in tracer avidities and sizes of lesions indicating favorable response.

**Fig. 2:**
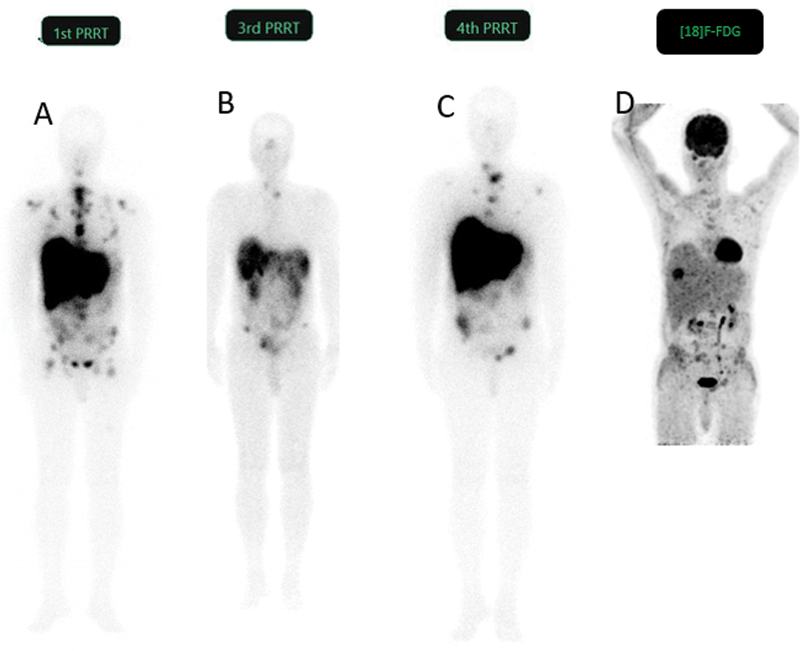
Post-[177Lu]Lu-DOTATATE therapy whole body (WB) images after 1st cycle of peptide receptor radionuclide therapy (PRRT) showed good tracer concentration in extensive liver and multiple skeletal disease ( **A** ). At 3rd cycle of PRRT, posttherapy WB image showed tracer uptake in a few liver and skeletal lesions ( **B** ). At 4th cycle of PRRT, WB image showed new radiotracer avid skeletal and liver lesions ( **C** ). The maximum intensity projection (MIP) image of [18F]-fluorodeoxyglucose (FDG)-positron emission tomography/computed tomography (PET/CT) at this time showed new tracer avid liver lesion (maximum standardized uptake value [SUVmax] 6.9) and skeletal lesions ( **D** ).

**Fig. 3:**
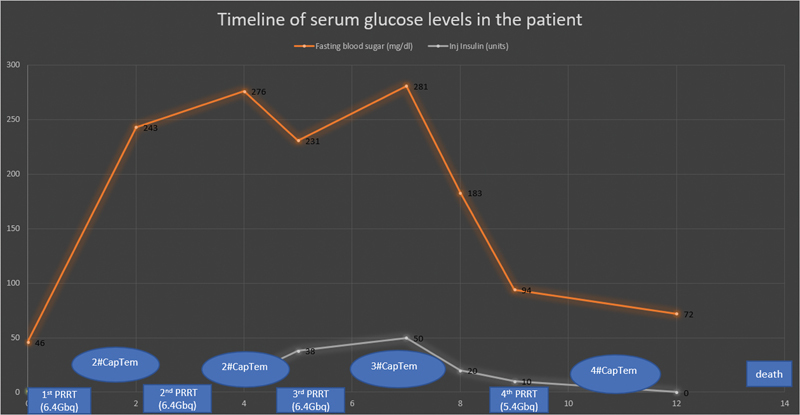
Graph of timeline of serum glucose levels since start of 1st cycle of peptide receptor radionuclide therapy (PRRT) to patient's demise. The graph depicts the unmasking of poor glycemic status in the patient of type 2 diabetes mellitus (DM) with insulinoma after first cycle of PRRT requiring oral antidiabetics (OAD) which showed no adequate glycemic control further necessitating insulin administration after 2 cycles of PRRT. During the course of management patient experienced loss of appetite and abdominal pain before 4th PRRT with decrease in the need for insulin administration and reduction in fasting blood glucose level suggesting progression of the insulinoma. The patient succumbed to urinary tract infection (UTI) and sepsis 14 months from the start of PRRT with no life-threatening hypoglycemic symptoms till 10 months after start of 1st cycle of PRRT.

During the course of therapy until cycle 3, patient symptoms of abdominal pain, loss of appetite, and hypoglycemia recurred, along with a decreased requirement for insulin administration. His tumor markers increased to 14,740 ng/dL for serum chromogranin A and 15.8 mg/24 h for urinary 5-HIAA, indicating progressive disease. He now received 4th cycle of PRRT. Post-[ ^177^ Lu]Lu-DOTATATE therapy scan showed tracer concentration in liver lesions and in new metastatic skeletal lesions. [ ^18^ F]FDG-PET/CT showed uptake in the liver and skeletal lesions and increased muscle uptake ( Fig. 2 ). The patient received 6.4 GBq of [ ^177^ Lu]Lu-DOTATATE per cycle for three cycles and 5.3 GBq during the last cycle at an interval of 8 to 12 weeks with cumulative activity of 24.5 GBq. The NETTER-1 trial advocated for an activity administration of 7.4 GBq per cycle, with a maximum of 4 cycles and a cumulative activity of 29.6 GBq. However, given the patient's extensive skeletal disease, we opted to administer a lower PRRT dose range of 5.3 to 6.4 GBq with palliative intent. This approach aimed to increase the number of cycles the patient could tolerate and to minimize toxicity. 3 After 4th cycle of PRRT, he presented with severe hypoglycemia requiring intravenous dextrose administration. He was then planned for molecular-targeted therapy but developed a urinary tract infection leading to sepsis and succumbed to disease. We also analyzed his fasting blood sugar (FBS) and insulin administration in relation to PRRT cycles. Initially, his FBS was 46 mg/dL with no requirement of OAD, post-one cycle of PRRT his FBS rose to 243 mg/dL suggesting poor glycemic control requiring OAD which did not control his blood glucose requiring administration of injection insulin. Subsequently, during progression of insulinoma, FBS showed a rapid fall, that required tapering insulin dose and eventually stopping before 4th cycle of PRRT as shown in Fig. 3 . The patient had progression-free survival (PFS) for 10 months after the first cycle of PRRT. However, he showed disease progression on clinical evaluation and posttherapy scintigraphic imaging (4th PRRT cycle) and subsequently died of complications. His overall survival (OS) was 26 months from the date of insulinoma diagnosis and 14 months from the first cycle of PRRT.

## Discussion

Malignant insulinoma is characterized by inappropriately high insulin secretion that present with severe and life-threatening hypoglycemic episodes that markedly impair the quality of life (QoL) of the patients. Among 121 patients with advanced insulinomas from the Surveillance, Epidemiology, and End Results registries around 40% had distant metastatic disease. 4 Metastatic insulinoma have poor prognosis with OS of 3.4 years and 10-year survival of approximately 30%. 5 The presence of Whipple's triad, symptoms, and signs of hypoglycemia that are reversed by administration of glucose are characteristic diagnostic features. DM with concurrent insulinoma is rare and diagnosis is easily missed as it can be misconstrued as improved glycemic control. The association between DM and insulinoma has been reported in literature. 6 Possible explanation includes patients with family history of diabetes show insulin resistance causing β islet cell autonomy. Insulinoma can mask detection of DM and a few cases were reported where the resection of the tumor led to DM and others only diagnosed in postmortem. In most of the available case reports, hypoglycemia was controlled with diazoxide and resection of tumor. 7

Metastatic insulinoma are rare in occurrence, so there is limited evidence regarding the best treatment option. The treatment usually follows the therapeutic algorithm of metastatic NET management, which has seen great advancement over the last decade. 8 First-line medical management includes SSAs for its antisecretory and antiproliferative effects with a good safety profile and diazoxide inhibits insulin secretion by activation on potassium channel and stimulates gluconeogenesis. First-line drugs for SSAs include octreotide and lanreotide. Injection octreotide LAR is a commonly prescribed medication and may interfere secretion of countercurrent hormones like glucagon and worsen hypoglycemia so short-acting SSA is to be given first as a challenge. Second-line drug for SSAs include pasireotide which has also shown favorable effects in metastatic insulinoma symptom control. 9 Diazoxide is a nondiuretic benzothiazide with response rates of 50 to 60% and effective rapid onset of action in approximately 50% of patients in controlling symptoms of hypoglycemia but can cause adverse effects like nausea, fluid retention, and hypertrichosis leading to noncompliance. 10 Cytotoxic chemotherapy can be considered in panNET with high burden, unresectable, progressive, and uncontrolled symptoms. Streptozocin plus doxorubicin with or without 5-fluorouracil has traditionally been used as first-line chemotherapeutic agent with response rates up to 41% in panNET in a retrospective study. 11 However, it has been replaced by less toxic CAPTEM. Molecular-targeted therapy including mammalian target of rapamycin (mTOR) inhibitors (everolimus) or tyrosine kinase inhibitors (sunitinib) showed improvement in PFS. Recent studies indicate everolimus as an effective treatment for metastatic insulinoma. Everolimus maintains body glucose homeostasis through Akt/mTOR pathway and acts independently as antisecretory and antineoplastic agents. 12 But, these agents often have adverse effects leading to requirement of dose reduction and at times dose suspension, only with modest clinical benefit.

PRRT in metastatic insulinoma for its antisecretory and antitumoral effects is performed with primary goal of disease control with improvement in OS and QoL. Zandee et al 13 showed that 67% of functioning panNET patients achieved symptomatic control despite radiological progression with PRRT. A recent retrospective analysis of 26 patients of metastatic insulinoma who received PRRT with average observation period of 21.5 months showed improvement in hypoglycemic symptoms in 81% of cases without life-threatening episodes of hypoglycemia. They found median OS of 19.7 months and PFS of 11.7 months after the PRRT. 14 Similar to our case, Kumar et al 15 reported a case of concomitant metastatic insulinoma and DM with no skeletal involvement treated with PRRT showed good sustained response of 6 years. Another reported case similar to ours by Magalhães et al 16 in a case series of metastatic insulinoma, reported skeletal involvement and [ ^18^ F]FDG uptake in hepatic lesions in a patient of insulinoma treated with three PRRT cycles at 16-week interval with cumulative activity of 17 GBq showed progressive clinical improvement with reduction of hypoglycemic episodes after first PRRT, eventually patient presented with clinical deterioration 15 months after PRRT and death at 17 months. Similarly, our patient with extensive FDG avid skeletal metastases showed partial response between treatment cycles and control of life-threatening hypoglycemic episodes for three cycles before the disease progressed on clinical evaluation and imaging.

## Conclusion

The prognosis for metastatic insulinoma is poor, and its occurrence in conjunction with DM is uncommon. Treatment options for this particular type of panNET are limited. PRRT can play an important role in the treatment of metastatic insulinoma with DM, leading to a decrease in life-threatening hypoglycemia and a favorable imaging response.
